# Clinicopathological correlates and prognostic significance of KRAS mutation status in a pooled prospective cohort of epithelial ovarian cancer

**DOI:** 10.1186/1746-1596-8-106

**Published:** 2013-06-25

**Authors:** Björn Nodin, Nooreldin Zendehrokh, Magnus Sundström, Karin Jirström

**Affiliations:** 1 Department of Clinical Sciences, Division of Pathology, Lund University, Skåne University Hospital, Lund 221 85, Sweden; 2 Department of Immunology, Genetics and Pathology, Uppsala University, Uppsala 751 85, Sweden

**Keywords:** KRAS mutation, Ovarian cancer, Prognosis

## Abstract

**Background:**

Activating KRAS mutations are common in ovarian carcinomas of low histological grade, less advanced clinical stage and mucinous histological subtype, and form part of the distinct molecular alterations associated with type I tumors in the dualistic model of ovarian carcinogenesis. Here, we investigated the occurrence, clinicopathological correlates and prognostic significance of specific KRAS mutations in tumours from 153 epithelial ovarian cancer (EOC) cases from a pooled, prospective cohort.

**Methods:**

KRAS codon 12,13 and 61 mutations were analysed by pyrosequencing in tumours from 163 incident EOC cases in the Malmö Diet and Cancer Study and Malmö Preventive Project. Associations of mutational status with clinicopathological and molecular characteristics were assessed by Pearson Chi Square test. Ovarian cancer-specific survival (OCSS) according to mutational status was explored by Kaplan-Meier analysis and Cox proportional hazards modelling. KRAS-mutation status was also analysed in 28 concomitantly sampled benign-appearing fallopian tubes.

**Results:**

Seventeen (11.1%) EOC cases harboured mutations in the KRAS gene, all but one in codon 12, and one in codon 13. No KRAS mutations were found in codon 61 and all examined fallopian tubes were KRAS wild-type. KRAS mutation was significantly associated with lower grade (p = 0.001), mucinous histological subtype (p = < 0.001) and progesterone receptor expression (p = 0.035). Kaplan-Meier analysis revealed a significantly improved OCSS for patients with KRAS-mutated compared to KRAS wild-type tumours (p = 0.015). These associations were confirmed in unadjusted Cox regression analysis (HR = 2.51; 95% CI 1.17-5.42) but did not remain significant after adjustment for age, grade and clinical stage. The beneficial prognostic impact of KRAS mutation was ony evident in tumours of low-intermediate differentiation grade (p = 0.023), and in a less advanced clinical stage (p = 0.014). Moreover, KRAS mutation was associated with a significantly improved OCSS in the subgroup of endometroid carcinomas (p = 0.012).

**Conclusions:**

The results from this study confirm previously demonstrated associations of KRAS mutations with well-differentiated and mucinous ovarian carcinomas. Moreover, KRAS-mutated tumours had a significantly improved survival in unadjusted, but not adjusted, analysis. A finding that merits further study is the significant prognostic impact of KRAS mutation in endometroid carcinomas, potentially indicating that response to Ras/Raf/MEK/ERK-targeting therapies may differ by histological subtype.

**Virtual slides:**

The virtual slide(s) for this article can be found here: http://www.diagnosticpathology.diagnomx.eu/vs/1788330379100147

## Background

Epithelial ovarian cancer (EOC) is the the leading cause of death from gynaecological malignancies and the fifth most common cause of cancer-related death in women [1]. Etiological factors involved in ovarian carcinogenesis remain poorly defined and the pitiable percentage of survival to incidence is related to cases being diagnosed in an advanced stage, most often stage III and IV, i.e. having metastatic spread to the lining of the abdomen or distant sites. Most patients relapse within 3 to 5 years despite harsh surgery and chemotherapy treatment [2]. Consequently, there is an urgent need to identify novel diagnostic, prognostic, and predictive biomarkers for development of improved personalized therapeutic regimens for ovarian cancer patients.

The KRAS (v-Ki-ras2 Kirsten rat sarcoma viral oncogene homolog) gene encodes the K-Ras protein, an important component of the tyrosine kinase signaling RAS/MAPK pathway. The K-Ras protein functions as a binary switch, binding GDP in its inactive state and GTP in the active, signal-emitting, state. To inactivate itself, the K-Ras protein interacts with GTPase-activating proteins (GAPs) and, when bound to GDP, it is not able to transmit signals to the cell nucleus. Missense point mutations in the KRAS gene abolish the GTPase function and, hence, lead to a constitutively activated protein that cannot turn itself off [3,4]. KRAS mutations, most commonly affecting codons 12 and 13, have been described in different types of solid tumors, with the highest proportion (up to 90%) reported in pancreatic cancer [5,6]. In recent years, the 2-type system for classification of EOC, proposed by Shih and Kurman in 2004, has become generally accepted [7]. According to this system, Type 2 cancers, encompassing the clinically more aggressive high-grade serous carcinomas, are defined by frequent mutations in p53 and BRCA1/2 genes, leading to genomic instability, while type 1 tumours, encompassing low-grade serous and endometroid carcinomas, clear cell, mucinous and transitional cell (Brenner) tumours, are characterized by common KRAS mutations [8,9]. KRAS mutations seem to occur early in the development of low-grade tumours, since they can be found in benign and borderline areas within the same neoplasm [10-14].

The aim of the present study was to examine the occurrence, clinicopathological correlates and prognostic significance of KRAS mutation status in tumours from 154 incident EOC cases from two prospective, population-based cohorts.

## Methods

### Patients

The study cohort is a pooled cohort consisting of all incident cases of EOC in the population-based prospective cohort studies Malmö Diet and Cancer Study (n = 101) [15] and Malmö Preventive Project Cohort (n = 108) [16] until Dec 31st 2007. Thirty-five patients participated in both studies, and archival tumor tissue could be retrieved from 154 (88,5%) of the total number of 174 cases. Cases were identified from the Swedish Cancer Registry up until 31 Dec 2006, and from The Southern Swedish Regional Tumour Registry for the period of 1 Jan - 31 Dec 2007. Histopathological, clinical and treatment data were obtained from the clinical and/or pathology records. Tumors were also re-evaluated regarding histological subtype and histological grade, using a three-tiered system, by a board certified pathologist (KJ). Information regarding clinical stage was obtained from the medical charts, following the standardized FIGO classification of tumor staging. Information on residual tumor after surgery was not available. Standard adjuvant therapy was platinum-based chemotherapy, from the 1990s given in combination with paclitaxel.

Information on vital status and cause of death was obtained from the Swedish Cause of Death Registry up until 31 Dec 2008. Follow-up started at date of diagnosis and ended at death, emigration or 31 Dec 2008, whichever came first. After a median follow-up of 2.65 years (range 0–21), 105 patients (68.2%) were dead and 49 (31.8%) alive. Patient-and tumour characteristics of the cohort have been described in detail previously [17-19]. Ethical permissions for the MDCS (Ref. 51/90), and the present study (Ref. 530/2008), were obtained from the Ethics Committee at Lund University.

### Tissue microarray construction and immunohistochemistry

TMAs were constructed as previously described [20]. Two 1 mm cores were taken from viable, non-necrotic tumor areas, when possible from both ovaries, and from concomitant peritoneal metastases (n = 33). Fallopian tubes with no evidence of histological disease were also sampled from 38 cases. Immunohistochemical expression of androgen receptor (AR), estrogen receptor (ER), progesterone receptor (PR), RNA-binding motif protein 3 (RBM3), minichromosome maintenance 3 protein (MCM3), Chek1, Chek2, Ki67 and special AT-rich sequence-binding protein1 (SATB1) was performed as previously described [17,21,22].

### Analysis of KRAS mutation status

The PyroMark Q24 system (Qiagen GmbH, Hilden, Germany) was used for pyrosequencing analysis of KRAS mutations on 1 mm formalin fixed paraffin-embedded tissue cores from benign-appearing fallopian tubes and from areas with >90% tumour cells in primary tumours. In brief, genomic DNA was extracted from tumour tissue in QIAamp MinElute spin columns (Qiagen) and the sequence of interest was amplified by PCR (Veriti 96 Well Fast Thermal Cykler, Applied Biosystems Inc., Foster City CA). Using therascreen KRAS Pyro Kit (Qiagen) KRAS mutations of codon 12, 13 and 61 were analysed and samples with a potential low-level mutation were reexamined in duplicates.

### Statistical analysis

Pearson’s Chi Square test was used for analysis of associations between KRAS mutation status and clinipathological and tumour biological characteristics. Kaplan-Meier analysis and log rank test were used to illustrate differences in ovarian cancer specific survival (OCSS) and overall survival (OS) according to KRAS mutation status in the full cohort and in strata according to differentiation grade, clinical stage and histological subtype. Cox regression proportional hazards models were used for estimation of hazard ratios (HRs) for death from ovarian cancer or overall causes according to KRAS mutation status in both uni- and multivariable analysis, adjusted for age, stage and differentiation grade. All calculations were performed using IBM SPSS Statistics Version 20 (SPSS Inc, Chicago, IL). All statistical tests were two-sided and a p value < 0.05 was considered statistically significant.

## Results

### Frequency of KRAS mutations in primary tumours and benign-appearing fallopian tubes

**Figure 1:**
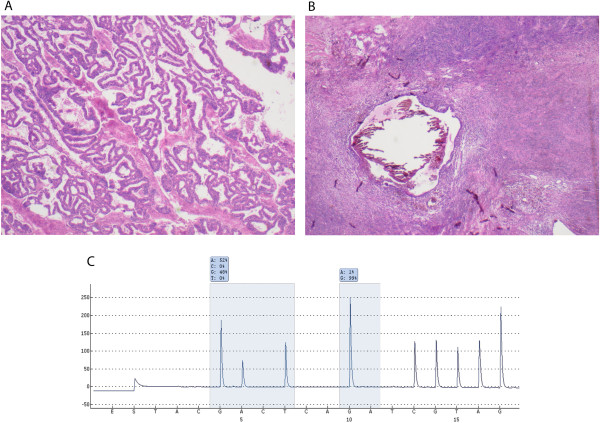
**KRAS-mutated endometroid cancer with concurrent endometriosis.** Haematoxylin and eosin stained sections of (**A**) an endometroid carcinoma with (**B**) concurrent endometriosis in the ovary and (**C**) a pyrogram showing a G12D (gly12 → asp12) mutation in codon 12.

KRAS mutation status could be assessed in 153/154 (99.3%) tumours. In the studied cohort of 153 EOC cases, 17 (11.1%) displayed mutations in the KRAS gene, 16 (10.5%) of which in codon 12 and 1 (0.7%) in codon 13. The most commonly found amino acid substitutions in codon 12 were G12D (gly12 → asp12) and G12V (gly12 → val12), representing 58% and 29% of mutations respectively (Table 1). No mutations in codon 61 were found in any of the tumours. All 28 successfully analysed benign-appearing fallopian tubes were KRAS wild-type. Notably, 13 (46.4%) of these fallopian tube samples were derived from patients diagnosed with serous carcinoma, all of which were also being KRAS wild-type (Table 2). Only 2/3 mucinous tumours with concomitantly sampled fallopian tubes harboured KRAS mutations (Table 2). Haematoxylin and eosin stained images from one case of endometroid carcinoma with concurrent ovarian endometriosis, and a pyrogram trace demonstrating a G12D (GGT → GAT) mutation in base 2 of codon 12, is shown in Figure 1.

**Table 1:** Distribution of specific KRAS mutations in 17 cases

**Mutation**	**Amino acid**	**N**	**%**	**%**
GGT → GAT	gly12 → asp12	5		29
GGT → TGT	gly12 → cys12	1		6
GGT → GTT	Gly12 → val12	5		29
GGT → GCT	gly12 → alanin12	2		12
GGT → AGT	gly12 → ser12	2		12
GGT → CGT	gly12 → arg12	1		6
GGT → GAC	gly13 → asp13	1		6
	**Total**	17		100

**Table 2:** Number of sampled fallopian tubes according to different histological subtypes and mutation status of the corresponding invasive tumours

**KRAS mutation status in invasive tumour**	**Number of sampled fallopian tubes according to histological subtype**					
	**Mucinuos**	**Serous**	**Endometroid**	**Clear cell**	**Undifferentiated**	**Total**
Wild-type	1	13	9	1	2	26
G12V	1					1
G12S	1					1
Total	3	13	9	1	2	28

### Associations of KRAS mutation status with clinicopathological and molecular parameters

Associations of KRAS mutation status with established clinicopathological and molecular characteristica are shown in Table 3. KRAS mutation was significantly associated with lower grade (p = 0.001), mucinous histological subtype (p = <0.001) and with PR expression (p = 0. 035), and a borderline significant inverse association with expression of Chek1 (p = 0.053). No associations were found between KRAS mutation status and age, clinical stage, or expression of ER, AR, or Chek2. Moreover, there were no significant associations between KRAS mutation status and expression of the proteins MCM3, RBM3, Ki67 or SATB1 (data not shown).

**Table 3:** Associations of KRAS mutation status with clinicopathological and molecular characteristics in 153 patients

**n (%)**	**KRAS wild type**	**KRAS mutated**	**P-value**
	**136(89%)**	**17(11%)**	
**Age**			
Mean	63.38	60.71	0.293
Median	62.00	62.00	
Range	47-83	49-69	
**Histological subtype**			
Mucinous	5(3.7)	7(41.2)	<0.001
Serous	87(64.0)	3(17.6)	
Endometroid	30(22.1)	5(29.4)	
Other	14(10.3)	2(11.8)	
**Differentiation grade**			
Well-moderate	36(26.5%)	11(64.7)	0.001
Poor	100(73.5)	6(35.3)	
**Clinical Stage**			
I	20(16.0)	6(40.0)	0.088
II	16(12.8)	2(13.3)	
III	70(56.0)	4(26.7)	
IV	19(15.2)	3(20.0)	
*Missing*	11	15	
**ER**			
≤10%	56/43.1)	11(64.7)	0.092
>10%	74(56.9)	6(35.3)	
*Missing*	6	0	
**PR**			
≤10%	111(84.1)	10(62.5)	0.035
>10%	21(15.9)	6(37.5)	
*Missing*	4	1	
**AR**			
≤10%	112(82.4)	13(76.5)	0.554
>10%	24(17.6)	4(23.5)	
*Missing*	0	0	
**Chek1**			
Low	36(28.8)	8(53.3)	0.053
High	89(71.2)	7(46.7)	
*Missing*	11	2	
**Chek2**			
Low	43(33.6)	9(56.2)	0.075
High	85(66.4)	7(43.8)	
*Missing*	8	1	

### Impact of KRAS mutation status on survival from EOC

**Figure 2:**
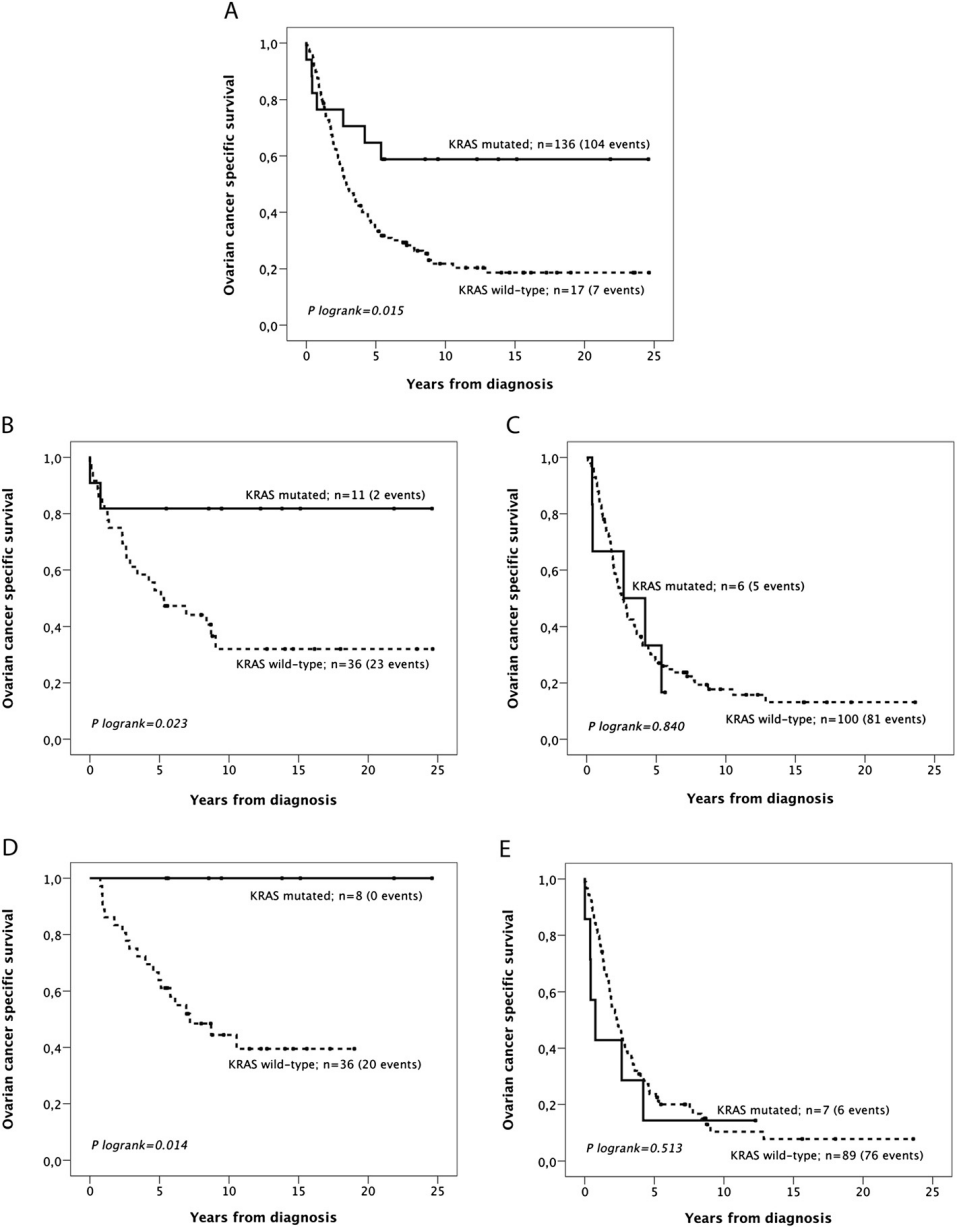
**Kaplan-Meier estimates of the prognostic impact of KRAS mutation status on survival from ovarian cancer in the full cohort and according to differentiation grade and clinical stage.** Kaplan-Meier analysis of ovarian cancer specific survival according to KRAS mutation status (**A**) in the full cohort, (**B**) in tumours of high-intermediate and (**C**) poor differentlation grade, in (**D**) FIGO Stage I-II tumours and (**E**) FIGO Stage III-IV tumours.

Kaplan-Meier analysis of the entire cohort (n = 153) revealed a significantly improved OCSS for patients with a KRAS mutation compared to KRAS wild-type patients (p = 0.015, Figure 2A). These associations were confirmed in univariable Cox regession analysis (HR = 2.51; 95% CI 1.17-5.42) but did not remain significant in multivariable analysis, adjusted for age, differentiation grade and clinical stage (HR = 1.46; 95% CI 0.61-5.42). Stratified analysis according to grade (well-moderate vs poorly differentiated) and stage (Figo I-II vs III-IV) revealed that the beneficial prognostic impact of KRAS mutation was only evident in tumours of low and intermediate differentiation grade (p = 0.023, Figure 2B) and tumours in a less advanced (FIGO I-II) clinical stage (p = 0.014, Figure 2D).

**Figure 3:**
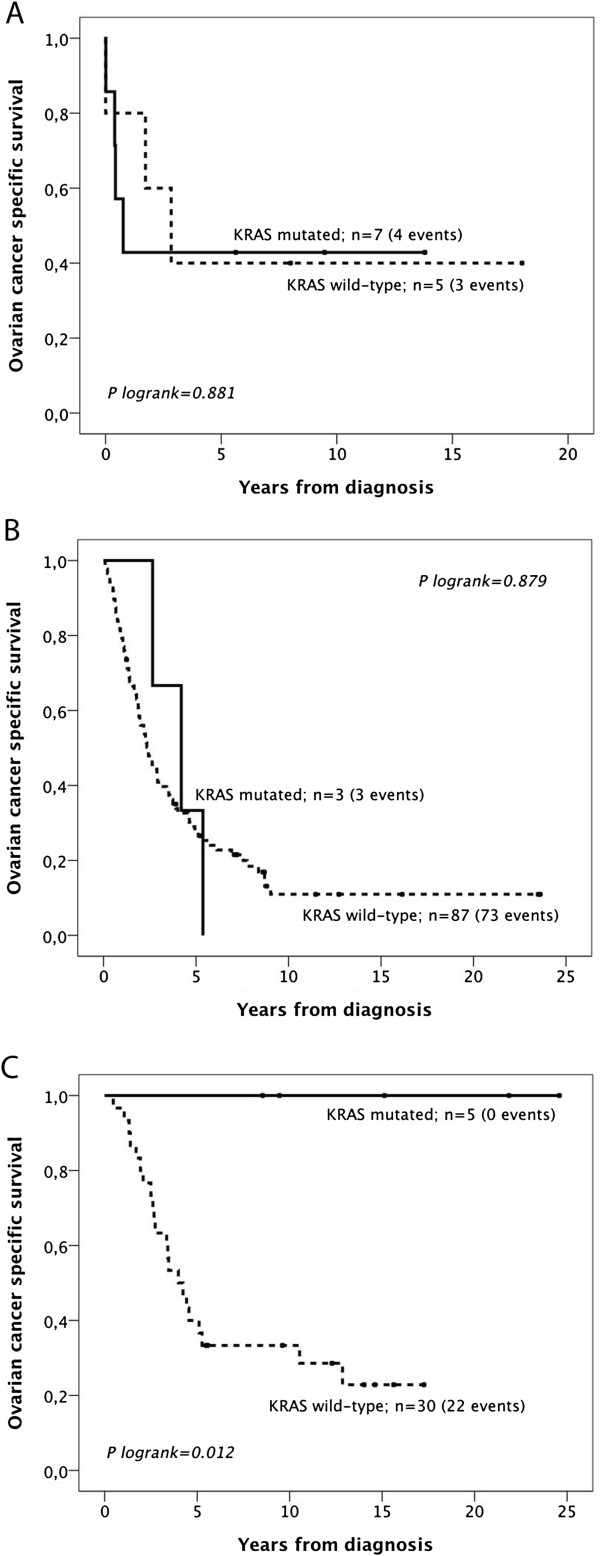
**Kaplan-Meier estimates of the prognostic impact of KRAS mutation according to histological subtype.** Kaplan-Meier analysis of ovarian cancer specific survival according to KRAS mutation status in (**A**) mucinous, (**B**) serous and (**C**) endometrial tumours.

Next, we examined whether the prognostic value of KRAS mutation status may differ according to histological subtype (Figure 3). This revealed that KRAS mutation was associated with a significantly improved OCSS in endometroid carcinomas (p = 0.012, Figure 3C), while KRAS mutation status was not a prognostic factor in mucinous (Figure 3A) or serous carcinomas (Figure 3B).

KRAS mutation status did not remain an independent prognostic factor in the subgroup analyses according to grade, stage and histological subtype, and there were no significant associations of KRAS mutation status with survival by grade and stage within different histological subtypes (data not shown). Overall survival rates were also compared in different subgroups and showed results in concordance with OCSS, (data not shown).

## Discussion

Epithelial ovarian cancer is a highly heterogenous disease with divergent clinical behaviour. This heterogeneity is not only reflected in the occurrence of different histological subtypes, but also in the tumourigenetic pathways [8,10,14,23-25]. While KRAS mutations have been demonstrated to signify Type 1 tumours, and hence, generally associated with a more favourable clinical course [10,13,26,27], few studies have investigated the prognostic value of KRAS mutation status in EOC. In this study, we have examined the occurrence, clinicopathological correlates and prognostic significance of KRAS mutation status in invasive tumours from 153 incident EOC cases from two prospective, population-based Swedish cohorts. The results demonstrate a frequency of KRAS mutations in line with previous reports [23,28,29]. All but one of the 17 (11%) cases with a KRAS mutation had mutations in codon 12, and one in codon 13. In resemblance with other studies [28,30] the most common amino acid substitutions in codon 12 were G12D (gly12 → asp12) and G12V (gly12 → val12). None of the cases harboured a mutation in codon 61, which is well in line with previous reports [10,11,28,31]. KRAS mutation status was also analysed in samples from benign-appearing fallopian tubes from 28 patients. All fallopian tube samples were KRAS wild-type, and KRAS mutations were only seen in two of three corresponding mucinous tumours. Although these findings do not allow any further conclusions regarding the putative origin of different EOC types, it would be of interest to analyse the occurrence of KRAS mutations in a larger set of matched fallopian tubes and invasive serous carcinomas, since the majority of these seem to derived from tubal epithelium [14]. Moreover, as this carcinogenetic pathway may proceed via the precursor lesion designated “serous intraepithelial tubal carcinoma (STIC) it would also be of interest to analyse the occurrence of specific mutations in this entity [32].

The significant association between KRAS mutation and mucinous histological subtype found here is well in line with previous reports [10,11,26,33].

The results from our study demonstrate that KRAS mutation is overall significantly associated with an improved survival in unadjusted analysis, but not in a multivariable model including age, differentiation grade and clinical stage, which is most likely explained by its association with a less aggressive tumour phenotype [14,34]. In line with previous findings, we found a strong association between KRAS mutations and more well-differentiated tumours [23,27]. Notably, all tumours in this study have been graded as well-, moderate- and poorly differentiated, according to the traditional three-tiered system. Recently, a two-tiered grading system into low-grade and high-grade tumours has been proposed for serous carcinomas, which seems to give more accurate prognostic and treatment predictive information for this category of tumours [35]. Since the overall proportion of tumours classified as being well-differentiated was rather low in this cohort, 8/154 (5.5%) in the full cohort and 2/90 (2.2%) among serous carcinomas, a dichotomized variable of well-moderately vs poorly differentiated grade was applied in the analyses. Nevertheless, although the two-tiered grading system may indeed be more informative about the nature and clinical behaviour of serous carcinomas, subgroup analysis did not reveal a differential prognostic impact of KRAS mutation status according to differentiation grade in serous carcinomas in our study.

We found no significant association between KRAS mutation status and clinical stage in this study, although the proportion of patients with FIGO Stage III-IV disease was higher in KRAS wild-type patients compared to KRAS mutated patients. Survival analysis stratified by clinical stage revealed that KRAS-mutation was associated with a favourable prognosis in tumours being in a less advanced, FIGO I-II, clinical stage, but not in FIGO Stage III-IV tumours, irrespective of histological subtype.

Of note, the fact that KRAS mutation status was only prognostic in more well-differentiated and less clinically advanced tumours may well be explained by the more frequent occurrence of KRAS mutations in these tumours, and should therefore be confirmed in larger cohorts before any further conclusions can be drawn.

Although being based on post-hoc analysis in a rather small subgroup, the finding of a significant prognostic value of KRAS mutation status in endometroid carcinomas is of potential interest, and has, to the best of our knowledge, not been demonstrated before. Of note, KRAS mutations have been suggested to distinguish endometroid carcinomas that are related to endometriosis from those that are not related to endometriosis [36] further indicating that KRAS status may indeed signify biologically and clinically relevant subgroups of endometroid carcinoma. Again, these findings need to be confirmed in a larger cohort of endometroid carcinomas, wherein the mutational status of concomitant endometriotic lesions should also be analysed. In this study, KRAS mutation status was not a prognostic factor in serous carcinomas, but, notably, the vast majority of tumours in this histological subgroup were KRAS wild-type.

A limitation to the present study is the lack of information on residual tumour after surgery, which is an important prognostic factor in EOC [37]. However, as KRAS mutation status did not provide any independent prognostic value, inclusion of this information in the multivariable model is not likely to have altered our findings.

In this study, we examined the associations between KRAS mutation status and several investigative factors, e.g. expression of hormone receptors AR, ER, PR, whereby an positive association was found between KRAS mutation and PR, but not ER or AR expression. High AR expression has previously been found to be an independent favourable prognostic factor in serous ovarian carcinoma in the here studied cohort, while ER and PR expression was not prognostic, neither in the full cohort nor in subgroup analysis according to histological type [17]. The inverse association between KRAS mutation and PR expression found here is in line with previous studies demonstrating a higher expression of ER and PR in low-grade serous carcinomas [38,39], although the number of KRAS-mutated serous tumours in our study was too low to make any direct comparisons [40]. Moreover, in another study, Hogdall et al. found that elevated expression of ER and PR, alone or in combination, was associated with an improved survival in a cohort of 773 Danish EOC patients [41].

Of note, KRAS mutational status was not significantly associated with expression of SATB1, a global gene regulator that has been demonstrated to be an independent factor of poor prognosis in high-grade tumours in the here examined cohort [21], as well as in several other cancer forms, e.g. breast [42] and colorectal cancer [43,44].

The borderline significant inverse association of KRAS mutation and high expression of Chek1 is well in line with the association of KRAS wild-type tumours being more genetically unstable [45]. DNA hyper-replication as a consequence of hyperproliferative oncogenic stimuli exposes the cell to replication stress [46] and triggers the activation of the checkpoint response [47,48]. Tumour cells often aquire defects in the checkpoint response in an early stage of tumour formation and deactivation of checkpoint proteins has been reported to cause genomic instability and predisposition to transformation into neoplastic cells [47-49].

## Conclusions

In this pooled prospective cohort of epithelial ovarian cancer, significant associations were found between KRAS mutations and mucinous histology, well differentiated tumours and positive progesterone expression. Patients with KRAS mutated tumours had a significantly improved survival in unadjusted analysis, and this beneficial impact of KRAS mutations on survival was only evident in patients having well and moderately differentiated tumours, and patients being diagnosed in a less advanced clinical stage. A finding of potential interest is the significant prognostic impact of KRAS mutation in endometroid carcinomas, but not in other histological subtypes. This association should be validated in future studies comprising larger patient cohorts, as the value of KRAS mutation status as a predictor of response to therapies targeting the Ras/Raf/MEK/ERK-pathway may differ by histological subtype.

## Abbreviations

KRAS: Kirsten rat sarcoma viral oncogene homolog; EOC: Epithelial ovarian cancer; OCSS: Ovarian cancer specific survival; OS: Overall survival; AR: Androgen receptor; ER: Estrogen receptor α; PR: Progesterone receptor; SATB1: Special AT-rich binding protein 1.

## Competing interests

The authors declare no conflict of interest.

## Authors’ contributions

BN carried out the pyrosequencing analyses, performed the statistical analyses, and drafted the manuscript. NZ and MS assisted with the pyrosequencing analysis and helped draft the manuscript. KJ conceived of the study and participated in its design and coordination and helped to draft the manuscript. All authors read and approved the final manuscript.
